# Metformin Inhibits TGF-β1-Induced Epithelial-to-Mesenchymal Transition via PKM2 Relative-mTOR/p70s6k Signaling Pathway in Cervical Carcinoma Cells

**DOI:** 10.3390/ijms17122000

**Published:** 2016-11-30

**Authors:** Keyan Cheng, Min Hao

**Affiliations:** Department of Obstetrics and Gynecology, The Second Hospital of Shanxi Medical University, Taiyuan 030000, China; chengkey@126.com

**Keywords:** metformin, mammalian target of rapamycin, epithelial-mesenchymal transition, PKM2

## Abstract

Background: Epithelial-to-mesenchymal transition (EMT) plays a prominent role in tumorigenesis. Metformin exerts antitumorigenic effects in various cancers. This study investigated the mechanisms of metformin in TGF-β1-induced Epithelial-to-mesenchymal transition (EMT) in cervical carcinoma cells. Methods: cells were cultured with 10 ng/mL TGF-β1 to induce EMT and treated with or without metformin. Cell viability was evaluated by CCK-8 (Cell Counting Kit 8, CCK-8) assay; apoptosis were analyzed by flow cytometry; cell migration was evaluated by wound-healing assay. Western blotting was performed to detect E-cadherin, vimentin, signal transducer and activator of transcription 3 (STAT3), snail family transcriptional repressor 2 (SNAIL2), phosphorylation of p70s6k (p-p70s6k) and -Pyruvate kinase M2 (PKM2) Results: TGF-β1 promoted proliferation and migration, and it attenuated apoptosis compared with cells treated with metformin with or without TGF-β1 in cervical carcinoma cells. Moreover, metformin partially abolished TGF-β1-induced EMT cell proliferation and reversed TGF-β1-induced EMT. In addition, the anti-EMT effects of metformin could be partially in accord with rapamycin, a specific mTOR inhibitor. Metformin decreased the p-p70s6k expression and the blockade of mTOR/p70s6k signaling decreased PKM2 expression. Conclusion: Metformin abolishes TGF-β1-induced EMT in cervical carcinoma cells by inhibiting mTOR/p70s6k signaling to down-regulate PKM2 expression. Our study provides a novel mechanistic insight into the anti-tumor effects of metformin.

## 1. Introduction

Cervical carcinoma is the second common gynecological carcinoma worldwide with more than 0.52 million new cases and 0.27 million deaths globally each year. Approximately 30% of cervical carcinoma patients will ultimately fail after surgery, radiotherapy, or chemotherapy treatment [1]. There is increasing evidence that Epithelial-to-mesenchymal transition (EMT) plays a prominent role in carcinoma tumorigenesis. The EMT enables carcinoma to invade and metastasize [2,3], induces cancer chemoresistance [4], and radioresistance [5,6], and has an immunoprotective effect [7]. Therefore, the EMT constitutes a major malignant propensity to cancer development and is a major obstacle to cure cancer.

During the EMT, epithelial cells undergo extensive genetic alterations, resulting in the loss of apical-basal polarity, the severing of cell-cell adhesion structures, and the degradation of basement membrane components [8]. The loss of E-cadherin is generally accepted as a hallmark of the EMT [9], which reduces cell-cell adhesion and destabilizes the epithelial architecture. This process is accompanied by increased expression of vimentin, which bestows a motile phenotype on cancer cells through changes in cellular architecture and cell-matrix interactions [10,11]. Snail, a transcription factor, acts as repressor of E-cadherin in response to TGF-β signaling [12], and has been linked to the induction of the EMT under different cellular contexts. A signal transducer and activator of transcription 3 (STAT3) is also involved in EMT by regulating the transcriptional regulators of E-cadherin [13]. Large studies indicated that alterations of EMT-related markers have been associated with metastatic disease and reduced survival, including cervical carcinoma [14,15].

Recent studies showed overexpression of pyruvate kinase M2 (PKM2) induced the epithelial-to-mesenchymal transition (EMT) and increased the metastatic potential of cancer cells [16]. PKM2 is an alternatively-spliced variant of the pyruvate kinase gene that is preferentially expressed during embryonic development and in cancer cells [17,18]. PKM2 regulates in the cancer-specific Warburg effect, which is responsible for the final rate-limiting step of glycolysis. Moreover, in cancer cells, PKM2 expression is associated with attenuated pyruvate kinase activity to meet the biosynthetic demands, which allows the diversion of glycolytic flux into the pentose phosphate pathway [18]. 

Metformin exerts its antitumorigenic effects through indirect mechanisms by increasing insulin sensitivity, inhibiting liver gluconeogenesis [19], and direct mechanisms involving activating AMP-activated protein kinase (AMPK), followed by inhibition of the mammalian target of the rapamycin (mTOR) pathway [20,21]. Moreover, metformin also plays a crucial role in modulating cell energy metabolism [22], and repressed the EMT through the mTOR signaling pathway [23]. Hosono et al. report that the mechanisms underlying the suppression on aberrant crypt foci formation of metformin are associated with the inhibition of the mTOR pathway [24]. Dann et al. reported that mTOR Complex1-S6K1 signaling is at the crossroads of obesity, diabetes, and cancer [25].

These mechanisms of metformin indicated that there likely is an antitumorigenic effect relationship between the mTOR pathway and PKM2 in various cancers. Moreover, the potential role of metformin in treating gynecologic oncology has been explored in a number of studies. A study reported that metformin inhibits βKlotho-related ERK1/2 signaling and AMPKα signaling to reverse the EMT in endometrial adenocarcinoma [26]. However, none of research involves the relationship of mTOR pathway and PKM2.

In this study, we investigate the role of metformin on inhibited TGF-β1-induced EMT in cervical carcinoma cells and explore the mechanisms that might be involved in tumorigenesis. Our data showed that the metformin reversed EMT. Metformin as the same anti-tumor effects as rapamycin, which decreased p-p70s6k and PKM2 expression. We infer that metformin is involved in mTOR/p70s6k/PKM2 signaling to promote cervical carcinoma resistance.

## 2. Results

### 2.1. Transforming Growth Factor Beta 1 (TGF-β1) Induces Epithelial-to-Mesenchymal Transition (EMT) in Cerivical Cancer Cells

**Figure 1:**
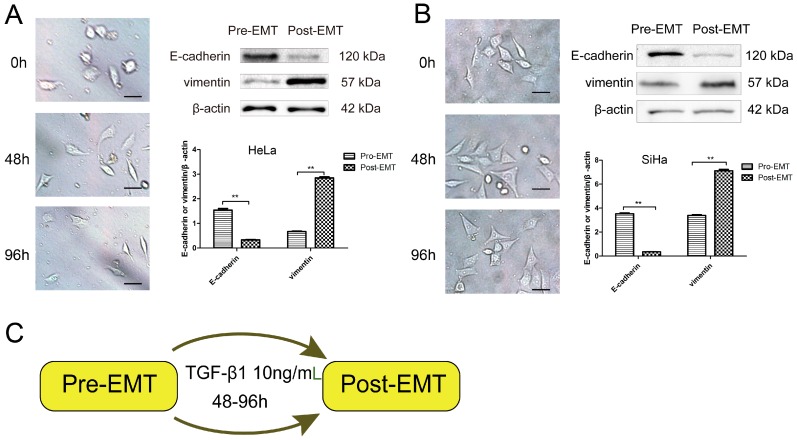
TGF-β1 induces EMT in cerivical cancer cells. (**A**,**B**) Photomicrographs of the morphological change in HeLa (**A**) and SiHa (**B**) cells. The number of hours indicates the period since EMT induction was initiated (scale bar, 50 μm). Western blot assays of E-cadherion, vimentin, and β-actin are shown in comparison with those in the pre-EMT condition; and (**C**) schematic representation of the procedure for EMT induction. The cells incubated for 48 h after seeding are defined as pre-EMT, and the cells cultured with 10 ng/mL TGF-β1 are defined as post-EMT. ** *p* < 0.01.

In order to determine whether TGF-β1 induced EMT, HeLa, and SiHa cells were incubated with 10 ng/mL TGF-β1 for 48 h based on Hamabe’ study [16]. The results obtained indicate that cells displayed an altered morphology, with flattened, stretched, and scattered fibroblast-like shapes. As shown in Figure 1, almost all HeLa (A) and SiHa (B) cells acquired spindle and fibroblastoid shapes with increased cell gaps. Moreover, protein levels of E-cadherin were abundantly expressed in the absence of TGF-β1 (pre-EMT). After these cells were stimulated by 10 ng/mL TGF-β1, E-cadherin expression was significantly decreased (post-EMT). In contrast, compared with the pre-EMT state, vimentin was increased (post-EMT), stimulated by TGF-β1 (Figure 1). In view of the changes in cell morphology and marker protein expression, these data indicated that EMT was induced when cervical carcinoma cells were stimulated with 10 ng/mL TGF-β1.

### 2.2. Metformin Inhibits the TGF-β1-Induced Proliferation, Migration, and Induces Apoptosis

**Figure 2:**
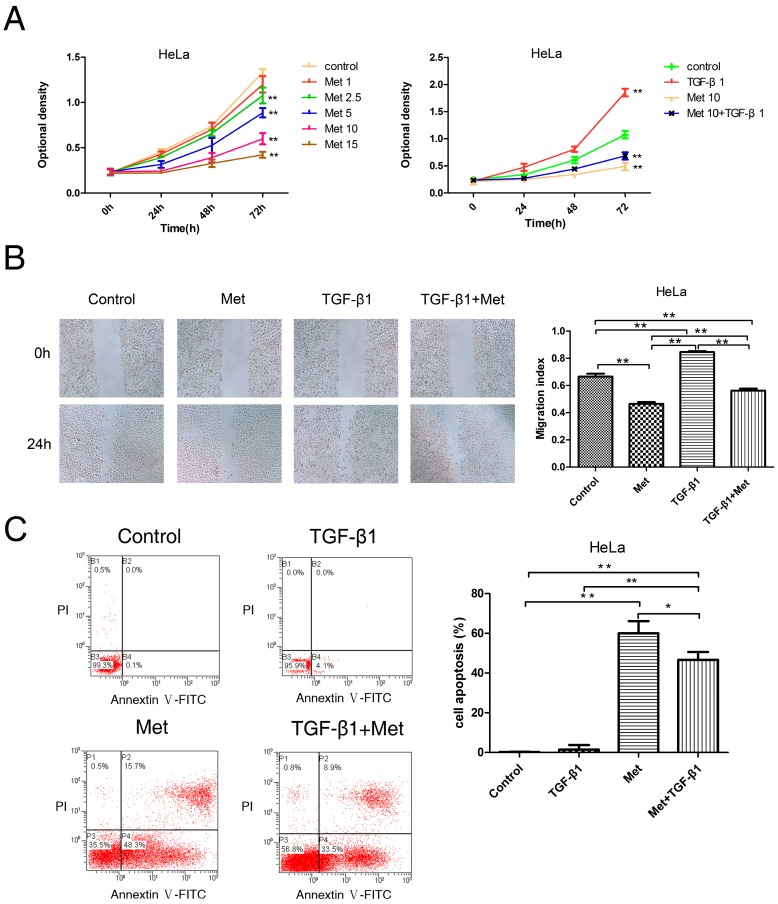
Metformin inhibits TGF-β1-induced proliferation, migration, and induces apoptosis in HeLa cells. (**A**) HeLa cells were treated with metformin (0, 1, 2.5, 5, 10 and 15 mM) or TGF-β1 with or without 10 mM metformin. Cell numbers were measured by CCK-8 assays at indicated times; (**B**) wound-healing assays. Representative images were obtained at 40× magnification. Graphs show the relative migration distance after 24 h incubation; (**C**) annexin V-FITC apoptosis assay. Cells were harvested and stained with Annexin V-FITC/PI (propidium iodide, PI), and cell apoptosis was analyzed using flow cytometry. Representative images are shown. TGF-β1: transforming growth factor β1; Met: metformin. * *p* < 0.05, ** *p* < 0.01.

**Figure 3:**
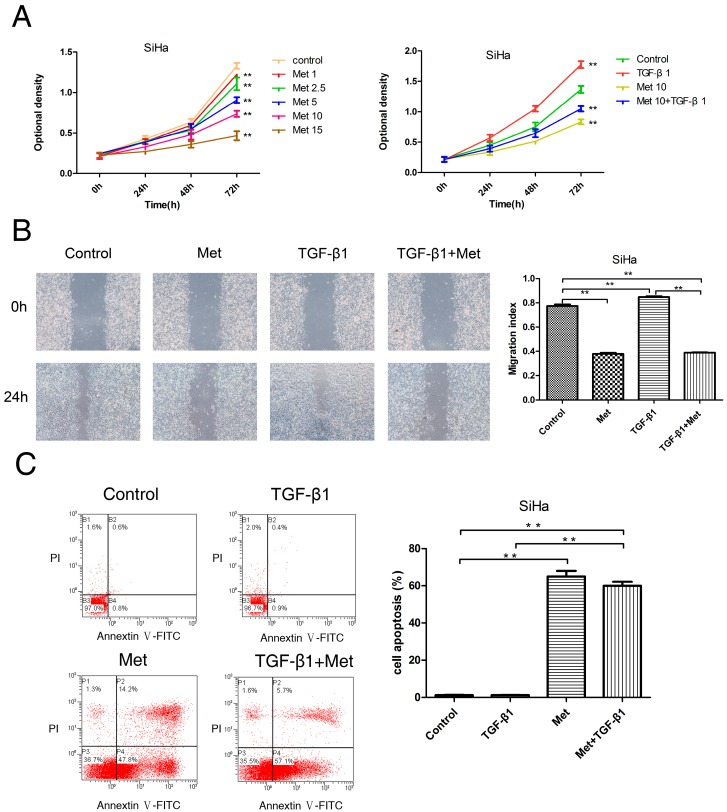
Metformin inhibits TGF-β1-induced proliferation, migration, and induces apoptosis in SiHa cells. (**A**) SiHa cells were treated with metformin (0, 1, 2.5, 5, 10, and 15 mM) or TGF-β1 with or without 10 mM metformin. Cell numbers were measured by CCK-8 assays at indicated times; (**B**) wound-healing assays. Representative images were obtained at 40× magnification. Graphs show the relative migration distance after 24 h incubation; (**C**) annexin V-FITC apoptosis assay. Cells were harvested and stained with Annexin V-FITC/PI (propidium iodide, PI), and cell apoptosis was analyzed using flow cytometry. Representative images are shown. TGF-β1: transforming growth factor β1; Met: metformin. ** *p* < 0.01.

To evaluate the potential anti-proliferative effect of metformin in cervical carcinoma cells, Cells were treated with 0, 1, 2.5, 5, 10, and 15 mM metformin, or 10 mM metformin with or without 10 ng/mL TGF-β1. The Cell Counting Kit-8 (CCK-8, Dojindo, Tokyo, Japan) assays were performed to determine the proliferation of cells. As shown in Figure 2A and Figure 3A, metformin led to a significant decrease in proliferation compared with untreated (control) cells, which inhibited the proliferation of HeLa (Figure 2A) and SiHa (Figure 3A) cell lines in a dose-dependent manner. In addition, treatment with TGF-β1 significantly increased the proliferation of both cell lines in comparison with treated metformin with or without TGF-β1 at 72 h, which was abolished by the addition of metformin. When comparing the metformin plus TGF group with the metformin group, cells treated with metformin plus TGF significantly proliferated faster than the metformin cells in SiHa cells, but not HeLa. It appears from the data in Figure 2 and Figure 3 that metformin only partially reversed the changes seen with TGF-β1.

The migration of cells were evaluated using wound-healing assays. Cells were treated with 10 mM metformin and with or without 10 ng/mL TGF-β1. Metformin significantly decreased the migration of cells compared with untreated (control) cells, and TGF-β1 significantly increased cell migration compared with untreated (control) cells in HeLa (Figure 2B) and SiHa (Figure 3B) cell lines at 24 h, which was abolished by the addition of metformin It was found that there is a statistical significance between the metformin group and the metformin with TGF-β1 group in HeLa cells (Figure 2B), but not in SiHa cells (Figure 3B). These data indicated that metformin could inhibit the migration ability of cells and reverse TGF-β1-induced EMT’s migration ability of cells.

To explore the potential effect of metformin for antagonzing the anti-apoptosis effect of TGF-β1 on cervical carcinoma cells by Annexin V-FITC and propidium iodide (PI) staining, we detected the effect of metformin with and without TGF-β1 on the apoptosis of HeLa (Figure 2C) and SiHa (Figure 3C) cells. The results obtained indicated that TGF-β1 induced a slight increase apoptosis compared with untreated cells in Hela cells (Figure 2C), but there was no statistical significance. Meanwhile, In SiHa (Figure 3C) cells treated with TGF-β1, the total apoptotic cells (early apoptotic + apoptotic) showed no effect compared to untreated cells. However, the apoptosis rate was increased by the addition of metformin in both cell lines. In addition, metformin with or without TGF-β1 exhibited a marked increase in apoptosis levels in both cell lines (Figure 2C and Figure 3C). These data indicated that the addition of metformin significantly abolished the TGF-β1-induced anti-apoptosis effects in both cell lines.

### 2.3. PKM2 Expression Is Required to Induce EMT

**Figure 4:**
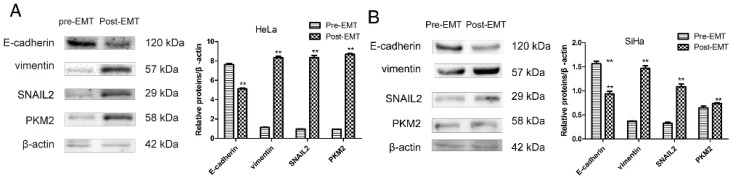
EMT condition stimulates an increase in PKM2. (**A**) HeLa and (**B**) SiHa cells were detected E-cadherin, vimentin, SNAIL2, and PKM2 expression by Western blot between pre-EMT and post-EMT state. Columns represent the average of at least three independent experiments; error bars represent the SD of the mean from triplicate results. ** *p* < 0.01.

Hamabe et al. reported that they used TGF-β1 to induce EMT in colorectal cancer cells. Then, siRNAs targeting PKM2 were designed to knockdown PKM2 in EMT conditions. They found that PKM2 knockdown failed to induce spindle-shaped morphological changes, and Western blot showed that PKM2 knockdown hindered E-cadherin loss and vimentin gain compared with the control [16]. To determine whether the EMT condition stimulates an increase in PKM2 compared with levels in the pre-EMT state. HeLa and SiHa cells were grown in a medium with 10 ng/mL TGF-β1. Consistent with this report, our results obtained showed that HeLa (Figure 1A) and SiHa (Figure 1B) cells changed morphology from epithelial to fibroblastic-like and spindle-shaped. E-cadherin expression was suppressed, whereas vimentin, and snail family zinc finger 2 (SNAIL2) expression were increased in the post-EMT condition (Figure 4). Moreover, PKM2 gene expression was induced in the EMT condition (Figure 4). The data indicated that the induction of EMT resulted in decreased E-cadherin expression, increased vimentin expression, and up-regulated PKM2 (Figure 4). These results confirmed that PKM2 expression was induced in the EMT condition.

### 2.4. mTOR/p70s6k Signaling Involved in Regulating PKM2 Expression in the EMT Condition

**Figure 5:**
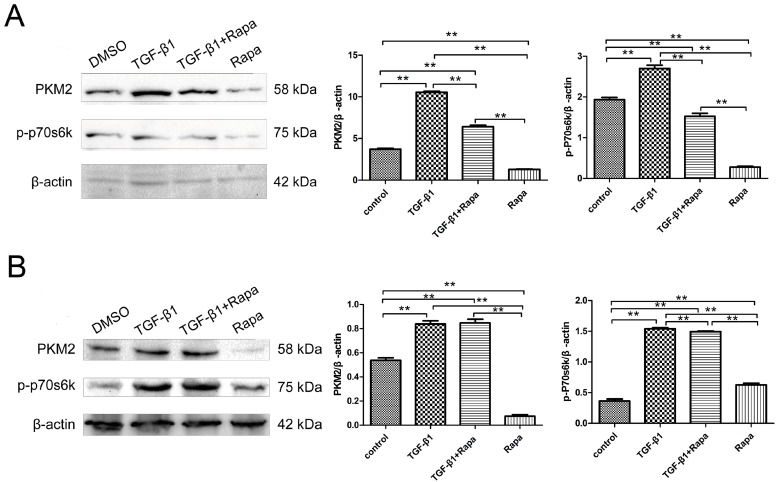
mTOR/p70s6k signaling involved in regulating PKM2 in the EMT condition. HeLa (**A**) and SiHa (**B**) cells were treated with TGF-β1, with or without rapamycin. Rapamycin was dissolved in DMSO and the same dose of DMSO was used as a control, and the p-p70s6k and PKM2 expressions were detected by Western blot. DMSO: dimethylsulfoxide; Rapa:rapamycin. ** *p* < 0.01.

To investigate whether the mTOR pathway affects PKM2 expression in the EMT condition, the mTOR pathway was inhibited by rapamycin (an mTOR inhibtior) to evaluate PKM2 (a critical glycolytic enzyme), and p70s6k (S6K1, a downstream effector of mTOR) expression. HeLa and SiHa cells were induced by TGF-β1 for 48 h, then were treated with or without 50 nM rapamycin for 24 h, respectively. Rapamycin was dissolved in dimethylsulfoxide (DMSO) and the same dose of DMSO was used as a control. As shown in Figure 5, TGF-β1 significantly increased the expression of PKM2 and phosphorylation of p70s6k. While, rapamycin, a specific mTOR inhibitor, inhibited the phosphorylation of p70s6k expression, ribosomal p70S6 kinase (S6K1) is one of main downstream mTOR effectors. Moreover, to investigate whether inhibition of mTOR/p70s6k signaling decreased PKM2 expression, which is one of the main downstream S6K1 effectors, rapamycin was added to cell cultures to inhibit the mTOR pathway. The results obtained indicate that inhibition of the mTOR pathway significantly decreased the expression of PKM2 and phosphorylation of p70s6k in HeLa (Figure 5A) and SiHa cells (Figure 5B). In addition, at a concentration of 50 nM rapamycin reversed TGF-β1-induced EMT expression by repressing PKM2 and p-p70s6k expressions in HeLa (Figure 5A) and SiHa cells (Figure 5B). These data indicated that the inhibition of EMT was through the PKM2 relative-mTOR/p70s6k signaling pathway in cervical carcinoma cells.

### 2.5. Metformin Reverses TGF-β1-Induced EMT Involved in mTOR/p70s6k/PKM2 Signaling Pathways in Cervical Carcinoma Cells

**Figure 6:**
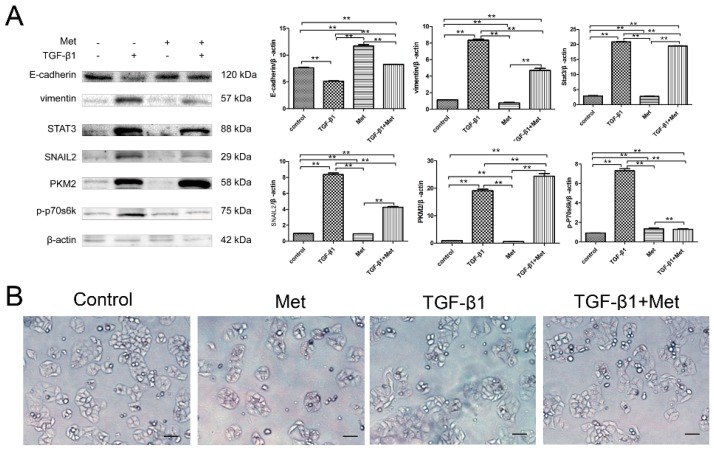
Metformin reverses TGF-β1-induced EMT in HeLa cells involving mTOR/p70s6k/PKM2 signaling pathways. (**A**) Cells were treated with TGF-β1, metformin, or both agents for 48 h. The protein expression levels of E-cadherin, vimentin, SNAIL2, STAT3, PKM2, p-p70s6k, and β-actin were detected by Western blot. β-actin was used as a loading control; and (**B**) the morphology of HeLa cells were treated with TGF-β1 and with or without metformin for 48 h. The cells were observed using phase contrast microscopy at 200× magnification. Scale bar: 50 μm. The data are presented as the mean ± SD of three replicates per group. TGF-β1: transforming growth factor β1; Met: metformin. ** *p* < 0.01.

**Figure 7:**
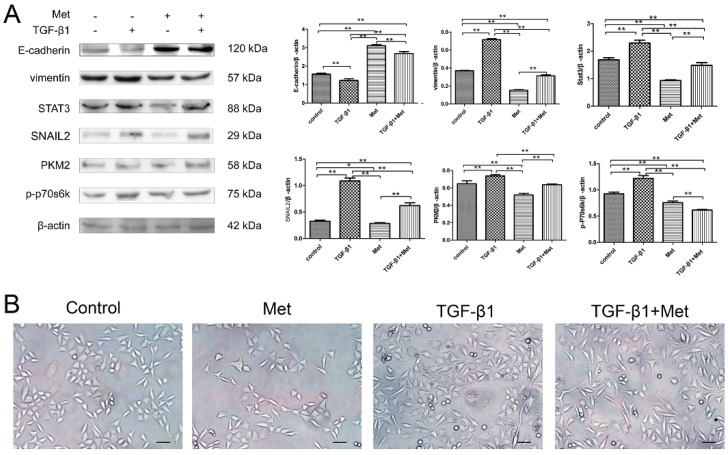
Metformin reverses TGF-β1-induced EMT in SiHa cells involving mTOR/p70s6k/PKM2 signaling pathways. (**A**) Cells were treated with TGF-β1, metformin, or both agents for 48 h. The protein expression levels of E-cadherin, vimentin, SNAIL2, STAT3, PKM2, p-p70s6k, and β-actin were presented by Western blot. β-actin was used as a loading control; and (**B**) the morphology of SiHa cells were treated with TGF-β1 with or without metformin for 48 h. The cells were observed using phase contrast microscopy at 200× magnification. Scale bar: 50 μm. The data are presented as the mean ± SD of three replicates per group. TGF-β1: transforming growth factor β1; Met: metformin. ** *p* < 0.01, * *p* < 0.05.

To determine the mechanism of metformin involved the regulation of EMT in cervical carcinoma, the expression of EMT-related markers were examined by Western blot. The results obtained revealed that 10 mM metformin caused an accumulation of E-cadherin, and decreased vimentin, STAT3, and SNAIL2 expression in HeLa (Figure 6A) and SiHa cells (Figure 7A). Moreover, TGF-β1 significantly decreased the expression of E-cadherin and increased the expressions of vimentin, STAT3, and SNAIL2 in HeLa (Figure 6A) and SiHa cells (Figure 7A). In addition, concentration of metformin reversed TGF-β1-induced EMT marker expression by repressing vimentin, STAT3, and SNAIL2 expressions and restoring E-cadherin expression in HeLa (Figure 6A) and SiHa cells (Figure 7A).

Next, we explored the possible signaling pathways that may be involved. As shown in Figure 6 and Figure 7, Western blots showed that metformin decreased the phosphorylation of p70s6k and down-regulated PKM2 levels in Hela (Figure 6A) and SiHa (Figure 7A) cells. TGF-β1 significantly increased the phosphorylation of p70s6k, which is the main downstream signaling intermediate of mTOR signaling. Simultaneously, TGF-β1 significantly up-regulated the expression of PKM2 in HeLa (Figure 6A) and SiHa cells (Figure 7A). While metformin reversed TGF-β1-induced EMT by repressing phosphorylation of p70s6k and PKM2 in HeLa (Figure 6A) and SiHa cells (Figure 7A). These results suggested that metformin reverses TGF-β1-induced EMT via the mTOR/p70s6k/PKM2 pathway.

Next, we examined the effect of metformin with or without TGF-β1 on the morphology of HeLa and SiHa cell lines. Metformin led to a significant decrease in proliferation in the both cells. Moreover, after stimulation with 10 ng/mL TGF-β1 for 48 h, both HeLa (Figure 6B) and SiHa cells (Figure 7B) cells became scattered, acquired a spindle-shaped morphology, and lost cell-cell contacts, which are characteristics of a mesenchymal-like morphology. Treatment with 10 mM metformin for 48 h abolished the TGF-β1-induced EMT morphological changes in HeLa and SiHa cells.The cells tended to aggregate and lose the spindle-shaped morphology.

## 3. Discussion

In this study, we demonstrated that metformin inhibited TGF-β1-induced EMT in proliferation, migration, and induced apoptosis. Our result suggested that metformin inhibits TGF-β1-induced EMT via the mTOR/p70s6k/PKM2 signaling pathway in cervical carcinoma cells.

Metformin is an anti-diabetic drug with potential anti-neoplastic action, which decreases the incidence and progression of multiple human cancers [27,28], and improves patients’ overall survival rate [29]. For example, in one study, ovarian or endometrial cancer patients with diabetes mellitus, who were being treated with metformin at the time of diagnosis, exhibited half the risk of mortality than that of the non-metformin-treated patients [29]. Moreover, metformin decreases hepatocellular carcinoma risk in a dose-dependent manner [30]. Several studies have also shown that metformin decreases cancer cell viability by inducing apoptosis in various cancer. Griss et al. reported that metformin inhibited cancer cell proliferation by suppressing mitochondrial-dependent biosynthetic activity [31], and metformin could induce breast cancer cell apoptosis [32]. In our study, we observed that metformin not only induced apoptosis and decreases cancer cell viability, but also reversed the anti-apoptosis effect of TGF-β1-induced EMT in cervical carcinoma cells. These data suggested the potential therapeutic implication of metformin for cervical carcinomas.

Recent studies indicated that metformin was a novel TGF-β suppressor with therapeutic potential for numerous diseases [33]. In our study, we used TGF-β1 to induce EMT and explore the possible mechanism of metformin that inhibited TGF-β1-induced EMT in cervical cancer. Moreover, metformin has been reported to inhibit EMT in lung adenocarcinoma [34], hepatocellular carcinoma [35], and inhibit mTOR signaling. PKM2 involved in tumorigenesis and affected the EMT situation. Silvestri et al. [36] reported that metformin induced apoptosis and down-regulated PKM2 in breast cancer cells. These studies showed that the mechanism of metformin may be associated with the following observations: (1) metformin inhibits mTOR activation by AMPK-dependence in different cancers [37,38,39]; (2) mTOR signaling regulates P70S6K signaling in cervical carcinoma cells [40]; (3) metformin induce intrinsic apoptosis via the inhibition of the HIF1α/PKM2 signaling pathway [41]; and (4) metformin is also a poisoner of mitochondria by impairing the function of complex I [42], leading to the increased aerobic glycolysis as compensation.

The mTOR pathway is a central regulator of glucose metabolism and glycolysis, and is important in the transcriptional program of glucose transporters and multiple rate-limiting glycolytic enzymes [43,44]. Meanwhile, the phosphorylation of p70 ribosomal S6 kinase 1 (S6K1), a downstream effector of mTOR, is modulated by the mTOR pathway [45]. Nobukini et al. reported that the activities of dS6k and S6K1 are regulated by the mammalian target of rapamycin (mTOR). They found the mechanisms regulating the mTOR/S6K1 signaling pathway will be fundamental in determining the mechanisms which control cell growth [46]. Montagne et al. reported that the deactivation of dS6K, the orthologue of mammalian S6K, is involved in slow overall growth rate and decreased cell size [47]. Consistent with these reports, in our study, we observed that metformin not only induced apoptosis and decreased cancer cell viability, but also abolished the TGF-β1 induced morphological changes through the mTOR/p70sk signaling pathway in cervical carcinoma cells.

Next, Hamabe et al. found that PKM2 plays a crucial role in the EMT development of cancer [16]. Recent studies also showed that PKM2 was an important glycolytic enzyme in the oncogenic mTOR-induced Warburg effect, in which hypoxia inducible factor-1α (HIF-1α) and c-Myc-hnRNP cascades are the transducers of mTOR regulation of PKM2 [48]. Sun et al. reported that mTOR up-regulation of pyruvate kinase isoenzyme type M2 plays a crucial role in aerobic glycolysis and tumor growth. PKM2 expression was augmented in mouse kidney tumors and consequent mTOR activation, and was reduced by mTOR suppression [48]. These studies illustrated that there was a correlation between mTOR/p70s6k/PKM2 signaling and EMT. Our data showed that TGF-β1 significantly increased the expression of PKM2 and phosphorylation of p70s6k, which means PKM2 and p70s6k are involved in EMT. Rapamycin, a mTOR inhibitor, inhibits the mTOR pathway, while p-p70sk6 and PKM2 were decreased. We prove that mTOR/p70s6k/PKM2 signaling is involved in EMT development.

**Figure 8:**
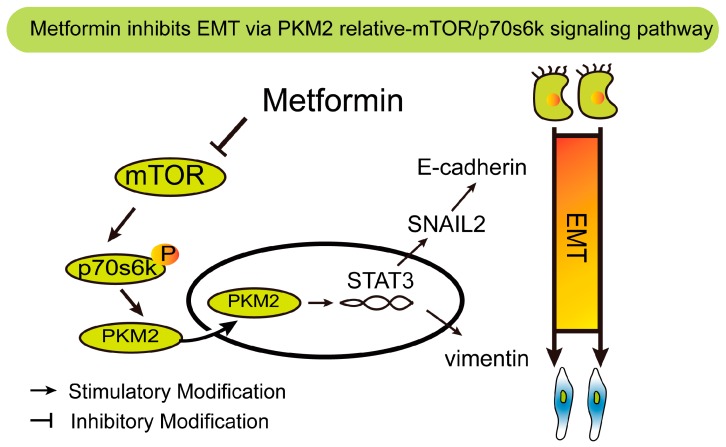
Schematic representation of metformin roles in TGF-β1-induced epithelial-to-mesenchymal transition in cervical carcinoma cells. EMT: Epithelial-to-Mesenchymal Transition; PKM2: Pyruvate kinase M2; P: STAT3: signal transducer and activator of transcription 3; SNAIL2: snail family transcriptional repressor 2; P: phosphorylation.

Based on these studies, we propose that metformin exerts its antitumorigenic effects and abolished TGF-β1-induced EMT through mTOR/p70s6k/PKM2 signaling in cervical carcinoma cells. In the present study, we showed that metformin significantly decreased cell proliferation and migration and reverses EMT in cervical carcinoma cells. More importantly, we demonstrated that mTOR/p70s6k/PKM2 signaling is the target of metformin. This claim is supported by the observation that mTOR activity is inhibited by addition of metformin in vitro. Moreover, we found that the role of metformin inhibit phosphorylation of p70s6k when cervical carcinoma was treated with metformin. In addition, our results indicated that metformin attenuates the phosphorylation of p70s6k and PKM2. Metformin reversed TGF-β1-induced epithelial-to-mesenchymal transition via the mTOR/p70s6k/PKM2 signaling pathway (Figure 8).

## 4. Materials and Methods

### 4.1. Cell Cultures and Treatments

The human cervical carcinoma cell lines HeLa (ATCC^®^ CRM-CCL-2™) and SiHa (ATCC^®^ HTB-35™) (ATCC, Rockville, MD, USA) were maintained in dulbecco’s modified eagle medium (DMEM)medium, supplemented with 10% fetal bovine serum (FBS) and 1% penicillin/streptomycin at 37 °C in a humidified environment with 95% air and 5% CO_2_. Rapamycin (PeproTech, Rocky Hill, NJ, USA), a specific mTOR inhibitor, was dissolved in dimethylsulfoxide (DMSO) at a stock concentration of 6.25 mM, and metformin (PeproTech, Rocky Hill, NJ, USA) was dissolved in phosphate-buffered saline (PBS) at a stock concentration of 50 mM. Both were stored at 4 °C. The cells were treated with 10 ng/mL TGF-β1 (PeproTech, Rocky Hill, NJ, USA) and with or without 10 mM metformin. The cells were collected for migration assay, CCK8 (CCK-8, Dojindo, Tokyo, Japan), and Western blot. The morphological changes of cells were observed under an inverted microscope.

### 4.2. Cell Viability Assay 

Cell viability was detected by cell counting kit-8 (CCK-8, Dojindo, Tokyo, Japan) assay. Cells were seeded into 96-well plates at 1 × 10^4^ cells/well and cultured overnight at 37 °C. At 24 h after seeding, the indicated concentrations of metformin, with or without TGF-β1, were added to each well and the cells were cultured for an additional 24, 48, and 72 h, respectively. At harvest time 10 μL of CCK-8 was added into each well and after one hour’s incubation, cell viability was determined by measuring the absorbance of the converted dye at 450 nm. The experiments were performed in triplicate.

### 4.3. Wound Healing Assay

A wound healing assay was performed to test cell migration. The cells were plated in six-well culture plates in complete culture medium and allowed to grow to 90% confluence. An injury line was made using a 2-mm-wide plastic pipette tip. After washing three times with PBS, the cells were cultured with fresh serum-free medium containing 10 ng/mL TGF-β1 with or without the indicated concentration of metformin for 24 h. Subsequently, the ability of the cells to migrate into the cleared section was observed using a microscope. The migration rate was quantified by (scratch distance at 0 h—scratch distance at 24 h)/scratch distance at 0 h. Representative images were obtained at 40× magnification. All experiments were repeated at least three times.

### 4.4. Annexin V-FITC Apoptosis Assay

Cells were seeded in six-well plates at 4 × 10^5^ cells/well and then treated with different concentrations of metformin, with or without 10 ng/mL TGF-β1 for 24 h. Apoptotic cells were detected by flow cytometry using an Annexin V-FITC kit according to the instructions.

### 4.5. Western Blot Analysis

The cells were harvested by centrifugation and washed with PBS. The cells were lysed in RIPA buffer containing protease inhibitors. Equal amounts of protein were separated by 10% sodium dodecyl sulfate-polyacrylamide gel electrophoresis and then transferred to Polyvinylidene Fluoride (PVDF) membranes by electroblotting. The membranes were blocked with 5% nonfat milk in Tris-buffered saline/0.1% Tween 20 for 1 h at room temperature and then incubated overnight at 4 °C with the primary antibodies. The anti-human E-cadherin, anti-human vimentin, anti-human SNAILl2, anti-human STAT3, and anti-human phospho-p70s6k primary antibodies were purchased from Cell Signaling Technology (Danvers, MA, USA). The anti-human β-actin primary antibodies and Horseradish Peroxidase (HRP)-conjugated goat anti-rabbit and HRP-conjugated goat anti-mouse secondary antibodies were purchased from ZSGB-BIO (Beijing, China). After incubation with the secondary antibody for 1 h at room temperature, the protein bands were detected using the ECL detection system (BD Biosciences, New York, NY, USA).

### 4.6. Statistical Analysis

The statistical analyses were performed using SPSS 19.0 (SPSS Inc., Chicago, IL, USA). The values are expressed as the means ± SD. Significant differences among groups were analyzed by one-way analysis of variance (ANOVA). An appropriate post-test has been applied for internal comparisons.

## 5. Conclusions

To our knowledge, this is the first study showing that metformin could reverse TGF-β1-induced EMT in tumor cells through mTOR/p70s6k/PKM2 pathways. Collectively, our data showed that TGF-β1induced proliferation and EMT, and metformin inhibited cell proliferation and reverseed EMT. The mechanism involved in the suppression of PKM2 activation was mediated by inhibiting mTOR/p70s6k signaling. Our data provides novel mechanistic insights into the antitumor effects of metformin.

## Abbreviations

TGF-β1	Recombinant transforming growth factor-β1
mTOR	Mammalian target of rapamycin
S6K1	Ribosomal p70 S6 kinase
PKM2	Pyruvate kinase M2
